# Clinical characteristics and laboratory parameters associated with the risk of severe COVID-19 in patients from two hospitals in Northeast Brazil

**DOI:** 10.1590/0037-8682-0119-2022

**Published:** 2022-09-26

**Authors:** Sara Larissa de Melo Araújo, Thiala Alves Feitosa, Vanessa Cardoso Pereira, Clara Caldeira de Andrade, Ana Tércia Paulo Silva, Lorena Viana de Andrade, Kamila Erika Ribeiro Lopes, Mirela Vanessa de Souza Sá, Carlos Dornels Freire de Souza, Anderson da Costa Armstrong, Rodrigo Feliciano do Carmo

**Affiliations:** 1 Universidade Federal do Vale do São Francisco, Programa de Pós-graduação em Ciências da Saúde e Biológicas, Petrolina, PE, Brasil.; 2 Universidade Federal do Vale do São Francisco, Programa de Pós-graduação em Biociências, Petrolina, PE, Brasil.; 3 Prefeitura de Petrolina, Petrolina, PE, Brasil.; 4 Universidade Federal do Vale do São Francisco, Colegiado de Ciências Biológicas, Petrolina, PE, Brasil.; 5 Universidade Federal do Vale do São Francisco, Colegiado de Farmácia, Petrolina, PE, Brasil.; 6 Universidade Federal de Alagoas, Programa de Pós-graduação em Saúde da Família, Maceió, Brasil.; 7 Universidade Federal do Vale do São Francisco, Colegiado de Medicina, Petrolina, PE, Brasil.

**Keywords:** Coronavirus, Predictors, Risk factors, SARS-CoV-2, Severity

## Abstract

**Background:**

Although most coronavirus disease 2019 (COVID-19) infections are mild, some patients have severe clinical conditions requiring hospitalization. Data on the severity of COVID-19 in Brazil are scarce and are limited to public databases. This study aimed to investigate the clinical and laboratory factors associated with the severity of COVID-19 in a cohort of hospitalized adults from two hospitals in Northeast Brazil.

**Methods:**

Patients over 18 years of age who were hospitalized between August 2020 and July 2021 with a confirmed diagnosis of COVID-19 were included. The patients were classified into two groups: moderate and severe. Clinical, laboratory and imaging parameters were collected and compared between the groups. A multivariate logistic regression model was used to determine the predictors of COVID-19 severity.

**Results:**

This study included 495 patients (253 moderate and 242 severe). A total of 372 patients (75.2%) were between 18 and 65 years of age, and the majority were male (60.6%; n = 300). Patients with severe disease had higher levels of leukocytes, neutrophils, platelets, neutrophil-to-lymphocyte ratio, monocyte-to-lymphocyte ratio, blood glucose, C-reactive protein, ferritin, D-dimer, aspartate aminotransferase, creatinine, and urea (p < 0.05). In multivariate logistic regression, the following variables were significant predictors of COVID-19 severity: leukocytes (odds ratio [OR] 3.27; 95% confidence interval [CI] 2.12-5.06), international normalized ratio (INR) (OR 0.22, 95% CI 0.14-0.33), and urea (OR 4.03; 95% CI 2.21-7.35).

**Conclusions:**

The present study identified the clinical and laboratory factors associated with the severity of COVID-19 in hospitalized Brazilian individuals.

## INTRODUCTION

Severe acute respiratory syndrome coronavirus 2 (SARS-CoV-2) is the virus responsible for causing coronavirus disease 2019 (COVID-19). The first case was reported in December 2019 in Wuhan, China^1^. Due to its rapid transmission, the disease has spread to several countries, and on January 30, 2020, it was declared a public health emergency of international interest. On March 11, 2020, the World Health Organization (WHO) classified COVID-19 as a pandemic^2^.

There have been 538,524,689 million cases worldwide, with 6,327,653 confirmed deaths^3^. In Brazil, the first confirmed case was reported in February 2020 in São Paulo in a man with a history of international travel^4^. Less than a month later, the country registered the first death. The disease quickly spread to large urban centers and reached the smallest and most distant municipalities, strongly influenced by the flow of people through air and road networks^5^ ^,^ ^6^. Brazil currently ranks third in the number of cases worldwide, with approximately 31 million confirmed cases and more than 667,000 deaths as of June 2022^3^. 

Most COVID-19 infections are mild, with symptoms or clinical features that generally include fever and cough, with recovery in 2-3 weeks^1^. However, some patients have severe clinical conditions requiring hospitalization, which can rapidly progress to acute respiratory distress syndrome, septic shock, refractory metabolic acidosis, coagulation disorders, multiple organ failure, and death^7^. Among the disease's most characteristic signs and symptoms are fatigue, cough, headache, myalgia, fever, and respiratory distress^8^ ^,^ ^9^. 

The following subgroups are considered at higher risk: having obesity, hypertension, diabetes, or heart disease; smokers; high-risk pregnant women; immunocompromised patients; patients with advanced-stage renal disease; people aged 60 years or over; and patients with malignant neoplasms, severe lung diseases, and chromosomal diseases^10^. Furthermore, some laboratory markers have been associated with disease severity, including troponin I, natriuretic peptide (BNP), D-dimer, C-reactive protein (CRP), albumin, interleukin-6, and ferritin^11^ ^-^ ^13^. 

Although factors related to the severity of COVID-19 have been described in several studies in European, Asian, and Latin American countries^14^ ^-^ ^16^, published studies from Brazil are limited to data available in public databases, with little clinical and laboratory information^17^ ^-^ ^19^. 

The identification of risk factors associated with the development of severe COVID-19 may provide subsidies for the early identification of risk groups, allowing better clinical management by the medical team, longer patient survival, and lower expenses related to the hospitalization and rehabilitation of these patients.

Therefore, this study aimed to investigate the clinical, laboratory, and imaging parameters associated with the severity of COVID-19 in a cohort of hospitalized adults from two hospitals in the city of Petrolina, located in the northeast region of Brazil.

## METHODS

### Design, population, and period

This observational cohort study was conducted in two hospitals in Petrolina, Northeast Brazil. Both hospitals serve as references for the treatment of COVID-19 in this region. The Monte Carmelo Field Hospital has 100 beds for intermediate care, and the University Hospital of the Federal University of Vale do São Francisco has 20 intensive care unit (ICU) beds dedicated to patients with COVID-19.

We included all patients older than 18 years who were hospitalized between August 2020 and July 2021 with suspected COVID-19. The diagnosis of COVID-19 was confirmed by reverse transcription followed by quantitative polymerase chain reaction (RT-qPCR) or a rapid swab test for viral antigens.

Patients included in the study were divided into 2 groups and classified according to the criteria established by the World Health Organization^20^, with modifications, as follows: I) Moderate cases: hospitalized, without requiring oxygen therapy or oxygen supplementation by means of a mask or nasal cannula; and II) Severe cases: hospitalized and required ventilatory support by non-invasive mechanical ventilation, high-flow devices, intubation, and mechanical ventilation, or mechanical ventilation with additional support (vasopressors, dialysis/hemodialysis, and extracorporeal oxygenation membrane). 

This study was approved by the Ethics and Research Committee of the Estácio de Sá University (CEP/UNESA, acronym in Portuguese) under the protocol CAAE:36613520.0.0000.5640 CEP/UNESA and was conducted in accordance with the provisions of the Declaration of Helsinki and the guidelines for good clinical practice.

### Data collection

Demographic, clinical, laboratory, and imaging parameters were obtained through data collection from electronic medical records.

Laboratory tests performed within 24h of admission were considered, including the following: complete blood count; activated partial thromboplastin time (APTT); D-dimer; aspartate aminotransferase (AST); alanine aminotransferase (ALT); total, direct, and indirect bilirubin; creatinine; ferritin; c-reactive protein (CRP); troponin T; urea; prothrombin time; international normalized ratio (INR); and gamma-glutamyl transferase (GGT). In addition, the neutrophil-to-lymphocyte ratio (NLR) and the monocyte-to-lymphocyte ratio (MLR) were calculated by dividing the absolute value of neutrophils by lymphocytes for NLR and the absolute value of monocytes by lymphocytes for MLR. The results of imaging tests such as radiography and computed tomography were also obtained.

We also collected data related to the period of hospitalization, such as maximum body temperature, highest respiratory rate, highest heart rate, lowest O2 saturation, mean blood pressure (mean blood pressure = diastolic blood pressure + [(systolic blood pressure − diastolic blood pressure)/3]), complications, drug treatment, outcome, length of hospital stay, and time between the onset of symptoms and hospitalization. 

### Statistical analysis

Data were analyzed using SPSS Statistics (version 22.0; SPSS, Inc., Chicago, IL, USA). GraphPad Prism version 8.0 (GraphPad, San Diego, CA, USA) was used to build the graphs. Categorical data were expressed as absolute frequencies and percentages. Continuous variables were presented as medians with interquartile ranges. The Kolmogorov-Smirnov test was used to verify the normal distribution of continuous variables. Comparisons between two groups were performed using the Student's t-test or the Mann-Whitney test for parametric or non-parametric approaches, respectively. Pearson's chi-squared test and Fisher's exact test were used for categorical variables.

To determine the predictors of severity associated with COVID-19, a multivariate logistic regression model using the backward method was applied. Clinical and laboratory variables collected at admission, with a p-value < 0.01 in univariate analysis, were included in the model. We decided to use a more conservative criterion for variable selection to obtain a simpler model with fewer parameters that can be more easily applied in clinical practice. The model was corrected according to sex and age. Logistic regression was followed by an a priori multicollinearity test using the tolerance test and variance inflation factor. The MissForest package in the R statistical program was used to impute laboratory data with up to 35% missing values. This program is based on machine learning and comprises an algorithm responsible for assigning lost data of multiple variables^21^.

Before insertion into the model, laboratory test results were transformed into binary data according to their respective reference values (Supplementary Table 1). Next, the odds ratio (OR) with a 95% confidence interval (CI) was estimated, and p-values < 0.05 were considered significant. Finally, the model's performance was evaluated using the area under the receiver operating characteristic curve (AUROC) analysis and 95% CI.

## RESULTS

### Clinical and demographic characteristics

**FIGURE 1:**
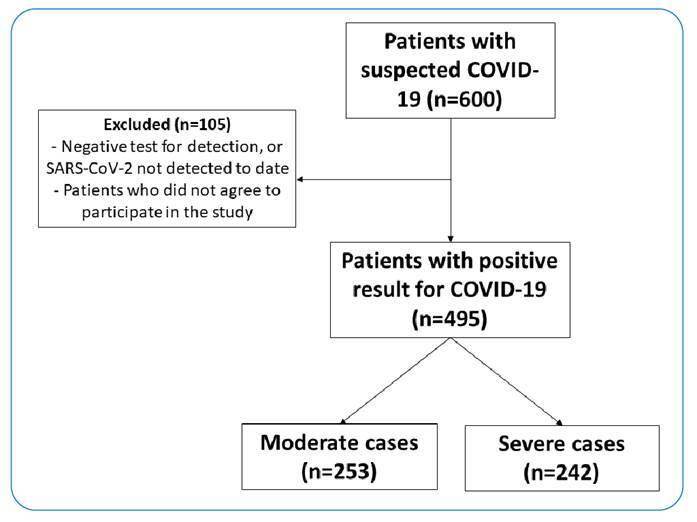
Flowchart of the patients included in the study and classified according to the severity criteria.

COVID-19: coronavirus disease 2019; SARS-CoV-2: severe acute respiratory syndrome coronavirus 2.

A total of 600 patients with suspected COVID-19 were recruited; 105 patients were excluded because they tested negative for COVID-19 or did not agree to participate in the study. In total, 495 patients were included in this study. The cases were subsequently classified as moderate (n = 253) or severe (n = 242) (Figure 1).

Table 1 describes the clinical and demographic characteristics of the patients included in the study, according to severity. Of the total cases, 372 patients (75.2%) were between 18 and 65 years of age, and the majority were male (60.6%; n = 300). The most frequently observed symptoms were dyspnea (74.9%; n = 371), dry cough (72.3%; n = 358), fever (66.9%; n = 331), and O2 saturation < 95% (51.9%; n = 257). The most common comorbidities were systemic arterial hypertension (48.7%; n = 241), diabetes mellitus (29.1%; n = 144), and obesity (23.6%; n = 117).

**TABLE 1:** Clinical and demographic characteristics of 495 patients with COVID-19 hospitalized in two hospitals in Northeast Brazil from August 2020 to July 2021.

	All patients (n=495)	Moderate (n=253)	Severe (n=242)	P-value
**Age**				
18 to 65 years old (n, %)	372 (75.2)	195 (77.1)	177 (73.1)	0.349
> 65 years (n, %)	123 (24.8)	58 (22.9)	65 (26.9)	
**Sex**				
Male (n, %)	300 (60.6)	149 (58.9)	151 (62.4)	0.462
Female (n, %)	195 (39.4)	104 (41.1)	91 (37.6)	
**Symptoms**				
Dyspnea (n, %)	371 (74.9)	196 (77.5)	175 (72.3)	0.213
Dry cough (n, %)	358 (72.3)	198 (78.3)	160 (66.1)	0.003
Fever (n, %)	331 (66.9)	183 (72.3)	148 (61.2)	0.010
O2 saturation < 95% (n, %)	257 (51.9)	132 (52.2)	125 (51.7)	0.928
Headache (n, %)	158 (31.9)	99 (39.1)	59 (24.4)	0.001
Muscle pain (n, %)	141 (28.5)	92 (36.4)	49 (20.2)	<0.0001
Anosmia/dysgeusia (n, %)	116 (23.4)	70 (27.7)	46 (19.0)	0.026
Asthenia (n, %)	107 (21.6)	72 (28.5)	35 (14.5)	<0.0001
Thoracic tightening (n, %)	78 (15.8)	38 (15.0)	40 (16.5)	0.712
Diarrhea (n, %)	71 (14.3)	45 (17.8)	26 (10.7)	0.029
Coryza/nasal congestion (n, %)	70 (14.1)	42 (16.6)	28 (11.6)	0.122
Sore throat (n, %)	32 (6.5)	16 (6.3)	16 (6.6)	1.000
Vomiting (n, %)	30 (6.1)	21 (8.3)	9 (3.7)	0.038
Nausea (n, %)	29 (5.9)	18 (7.1)	11 (4.5)	0.254
Body pain (n, %)	24 (4.8)	17 (6.7)	7 (2.9)	0.059
General malaise (n, %)	20 (4.0)	13 (5.1)	7 (2.9)	0.256
Abdominal pain (n, %)	16 (3.2)	10 (4.0)	6 (2.5)	0.449
Joint pain (n, %)	5 (1.0)	5 (2.0)	0	0.062
Comorbidities				
Systemic arterial hypertension (n, %)	241 (48.7)	110 (43.5)	131 (54.1)	0.019
Diabetes mellitus (n, %)	144 (29.1)	60 (23.7)	84 (34.7)	0.008
Obesity* (n, %)	117 (23.6)	51 (20.2)	66 (27.3)	0.072
Chronic kidney disease (n, %)	19 (3.8)	3 (1.2)	16 (6.6)	0.002
Chronic obstructive pulmonary disease (n, %)	18 (3.6)	9 (3.6)	9 (3.7)	1.000
Chronic heart disease (n, %)	15 (3.0)	4 (1.6)	11 (4.5)	0.067
Asthma (n, %)	13 (2.6)	6 (2.4)	7 (2.9)	0.784
Neoplasm (n, %)	7 (1.4)	1 (0.4)	6 (2.5)	0.063
Chronic lung disease (n, %)	5 (1.0)	4 (1.6)	1 (0.4)	0.373
**Risk Factors**				
Smoking history (n, %)	119 (24.0)	60 (23.7)	59 (24.4)	0.916

* Obesity was defined based on the impressions of the medical team.

Dry cough, fever, headache, muscle pain, anosmia/dysgeusia, asthenia, diarrhea, and vomiting were most often reported by individuals with moderate disease (p < 0.05). Regarding comorbidities, diabetes, hypertension and chronic kidney disease were related to the severity of COVID-19 (p < 0.05) (Table 1).

### Clinical data on hospitalization

Table 2 describes the clinical data on hospitalization, treatment, and outcomes, including moderate and severe cases. It was observed that severe cases had higher values of body temperature, respiratory rate, heart rate, and a fraction of inspired oxygen when compared to the group of moderate cases (p < 0.0001). In addition, the O2 saturation levels and mean arterial pressure were lower in the severe group (p < 0.0001).

**TABLE 2:** Clinical data on hospitalization, treatment, and outcome of patients with COVID-19 in Brazil, 2020 to 2021.

	All patients (n=495)	Moderate (n=253)	Severe (n=242)	P-value
**Clinical signs upon hospitalization**				
Highest heart rate, median (IQR)	104 (93-122)	97.5 (88-105.75)	119 (100-137.75)	<0.0001
Lowest O2 saturation (%), median (IQR)	90 (88-93)	92 (89.5 - 93.0)	89 (85-92)	<0.0001
Mean blood pressure (mmHg), median (IQR)	73 (69.5 - 87.00)	80 (73-89)	71 (73-89)	<0.0001
Maximum body temperature (°C), median (IQR)	37.45 (36.60-38.30)	37 (36.4-37.7)	38 (37.10-38.70)	<0.0001
Fraction of inspired oxygen (%), median (IQR)	32 (21-50)	24 (21-32)	50 (35-60)	<0.0001
Highest respiratory rate, median (IQR)	26 (23-32)	24 (22-28)	29 (25-36)	<0.0001
**Image changes**				
Bilateral alveolar infiltrate (n, %)	31/134 (23.1)	16/55 (29.1)	15/79 (19.0)	0.053
Frosted glass pattern (n, %)	31/134 (23.1)	11/55 (20.0)	20/79 (25.3)	1.000
Pneumothorax (n, %)	12/134 (9.0)	0/55	12/79 (15.2)	0.005
Unilateral alveolar infiltrate (n, %)	8/134 (6.0)	6/55 (10.9)	2/79 (2.5)	0.452
Cardiomegaly (n, %)	3/134 (2.2)	2/55 (3.6)	1/79 (1.3)	0.608
Pleural effusion (n, %)	3/134 (2.2)	1/55 (1.8)	2/79 (2.5)	0.272
Interstitial infiltrate (n, %)	2/134 (1.5)	0/55	2/79 (2.5)	1.000
Complete pulmonary opacification (n, %)	2/134 (1.5)	1/55 (1.8)	1/79 (1.3)	1.000
**Oxygen support**				
Mechanical ventilation (n, %)	186/437 (37.6)	0/195	186/242 (76.9)	<0.0001
Nasal catheter (n, %)	174/437 (39.8)	174/195 (89.2)	0/242	
Non-invasive ventilation (n, %)	56/437 (11.3)	0/195	56/242 (23.1)	
Non-rebreathing mask (n, %)	21/437 (4.8)	21/195 (10.8)	0/242	
**Complications**				
Acute kidney injury (n, %)	80 (16.2)	3 (1.2)	77 (31.8)	<0.0001
Shock (n, %)	61 (12.3)	1 (0.4)	60 (24.8)	<0.0001
Cardiorespiratory arrest (n, %)	44 (8.9)	0	44 (18.2)	<0.0001
Sepsis (n, %)	44 (8.9)	1 (0.4)	43 (17.8)	<0.0001
Myocardial failure (n, %)	7 (1.4)	0	7 (2.9)	0.006
Myocardial infarction (n, %)	1 (0.2)	0	1 (0.4)	0.489
**Drug treatment**				
Use of anticoagulants (n, %)	490 (99.0)	249 (98.4)	241 (99.6)	0.373
Corticosteroid use (n, %)	474 (95.8)	235 (92.9)	239 (98.8)	0.001
Use of bronchodilators (n, %)	424 (85.7)	227 (89.7)	197 (81.4)	0.010
Antibiotic use (n, %)	232 (46.9)	77 (30.4)	155 (64.0)	<0.0001
Vasopressor medication (n, %)	190 (38.4)	33 (13.0)	157 (64.9)	<0.0001
Antiparasitic use (n, %)	55 (11.1)	26 (10.3)	29 (12.0)	0.570
**Length of stay, median (IQR)**	6 (3.00-12.00)	4 (2.00-5.25)	12 (6.00-20.00)	<0.0001
**Onset of symptoms to date of admission (days), median (IQR)**	9.00 (6.00-11.00)	9.00 (7.00-11.00)	9.00 (6.00-11.00)	0.942
Outcome				
Recovery (n, %)	389 (78.6)	241 (95.3)	148 (61.2)	<0.0001
Death (n, %)	87 (17.6)	0	87 (36.0)	
Transfer to another hospital (n, %)	19 (3.8)	12 (4.7)	7 (2.9)	

**IQR:** interquartile range.

Of the 134 (27.0%) patients who underwent imaging tests, bilateral alveolar infiltrate (23.1%; n = 31) and the ground-glass pattern (23.1%; n = 31) were the most prevalent. Among the findings, only pneumothorax was significantly more frequent in the severe COVID-19 group (p = 0.005) (Table 2).

Regarding oxygen support, the severe group predominantly used invasive mechanical ventilation (76.9%) and non-invasive ventilation (23.1%), whereas the moderate group used mainly nasal catheters (89.2%) and non-rebreathing masks (10.8%) (Table 2).

During hospitalization, a greater number of complications were observed in severe cases. Cardiorespiratory arrest, sepsis, shock, myocardial failure, and acute kidney injury were significantly more common in the severe group when compared to the moderate COVID-19 group (p < 0.01) (Table 2).

Regarding the use of prescription drugs, in the total number of patients, anticoagulants (99.0%; n = 490), corticosteroids (95.8%; n = 474), bronchodilators (85.7%; n = 424), and antibiotics (46.9%; n = 232) were the most used (Table 2).

Regarding the length of hospital stay, a median of 6 days was observed for the total number of hospitalized patients, with a longer duration in the severe group than in the moderate group (12 days vs. 4 days, p < 0.0001). There was no significant difference between the symptom onset and hospitalization date between the two groups (p = 0.942). In the severe COVID-19 group, 36.0% (n = 87) of patients died, while in the moderate group, 95.3% (n = 241) of patients recovered and 4.7% (n = 12) were transferred (p < 0.0001) (Table 2).

### Laboratory data

**FIGURE 2:**
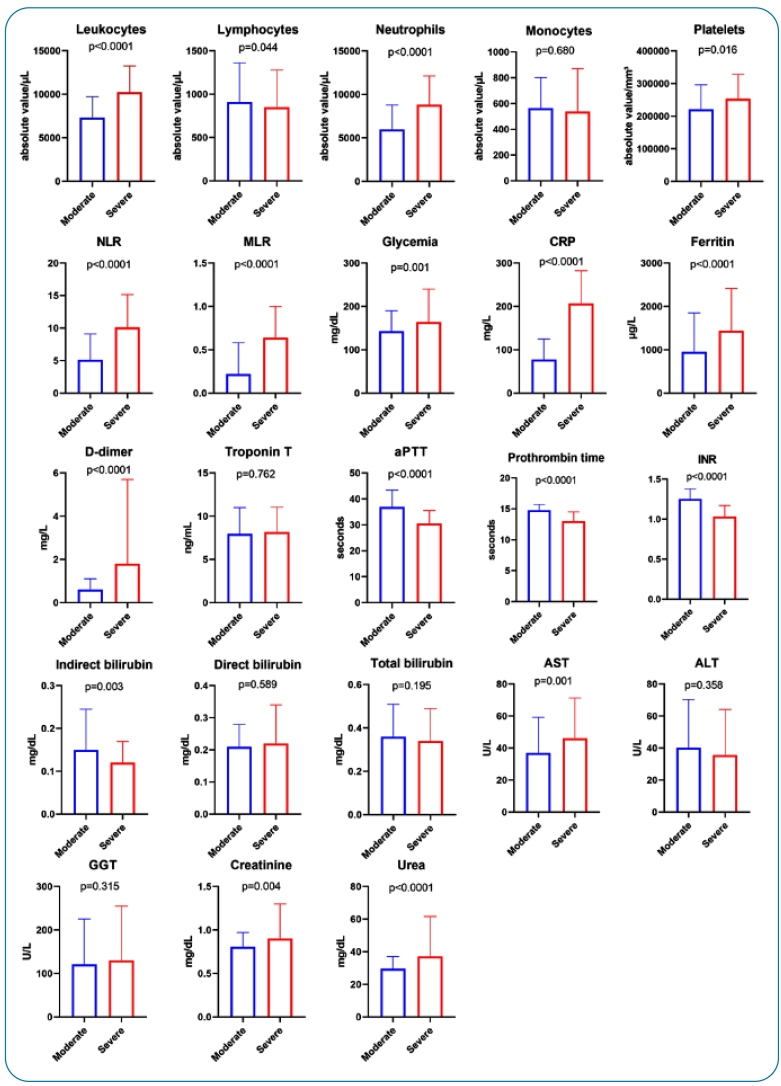
Laboratory data of COVID-19 patients associated with severity. Results were presented as the median and interquartile range (IQR). Mann-Whitney test was used for comparison between groups.

Regarding laboratory findings, it was observed that severe patients had higher levels of leukocytes, neutrophils, platelets, NLR, MLR, blood glucose, CRP, ferritin, D-dimer, AST, creatinine, and urea (p < 0.05). In contrast, patients with moderate COVID-19 had higher levels of lymphocytes, APTT, prothrombin time, INR, and indirect bilirubin (p < 0.05) (Figure 2).

### Multivariate logistic regression

Multivariate logistic regression using the backward method was used to identify the predictors of severity associated with COVID-19. The model initially included the following variables: glucose, leukocyte, C-reactive protein (CRP), ferritin, INR, AST, and urea levels. The other variables were excluded because they had p-value > 0.01, had more than 35% absent values, or were redundant. After excluding the variables using the backward regression method and adjusting according to sex and age, the following variables remained in the model: leukocytes > 10,000 cells/μl (p < 0.0001; OR 3.27; 95% CI 2.12 to 5.06), INR > 1.2 (p < 0.0001; OR 0.22; 95% CI 0.14 to 0.33), and urea > 48.5 mg/dL (p < 0.0001; OR 4.03; 95% CI 2.21 to 7.35) (Table 3). The AUROC was 0.78 (95% CI 0.74 to 0.82). 

**TABLE 3:** Multivariate logistic regression for risk factors for severity in patients with COVID-19.

	Multivariate analysis	
	OR (95% CI)*	p-value
Leukocytes (> 10,000 cells/μl)	3.27 (2.12-5.06)	<0.0001
INR (> 1.2)	0.22 (0.14-0.33)	<0.0001
Urea (> 48.5 mg/dL)	4.03 (2.21-7.35)	<0.0001

**INR:** international normalized ratio. *Corrected for age and sex.

## DISCUSSION

Due to the challenging behaviors of COVID-19, understanding the reasons contributing to aggravation is extremely important. They are a priority when facing a rapidly spread public health emergency such as this one.

Early identification of risk factors can contribute to better care for patients with a greater chance of developing severe disease. Thus, this study analyzed the risk factors associated with the severity of COVID-19 in hospitalized patients in Northeast Brazil.

Diabetes, hypertension, and chronic kidney disease were associated with severe COVID-19 compared to patients with a moderate form of the disease. These findings are consistent with previous results that demonstrated a higher risk of hospitalization in older male patients with comorbidities^16^ ^,^ ^22^ ^,^ ^23^. A meta-analysis including 77 studies and 38,000 patients demonstrated an increased risk of death in patients with the following risk factors: age > 60 years (summary relative risk [SRR] = 3.6), male sex (SRR = 1.3), history of smoking (SRR = 1.3), COPD (SRR = 1.7), heart disease (SRR = 2.1), chronic kidney disease (SRR = 2.5), hypertension (SRR = 1.8) and diabetes (SRR = 1.5)^16^. A study in the United States, including 10,131 veterans, showed that older age, male sex, diabetes, hypertension, chronic kidney disease, cirrhosis, and alcohol dependence were associated with the risk of hospitalization^24^. Fever, dyspnea, nausea, and diarrhea were significantly associated with the risk of hospitalization^24^. The present study's findings corroborate previous work carried out by our group using 59,695 cases of COVID-19 in Northeast Brazil registered in a public database, which demonstrated a higher risk of death associated with age, sex, and presence of comorbidities^25^. Other Brazilian studies have reported similar results^17^ ^-^ ^19^.

In total, 76.9% of severe patients received invasive mechanical ventilation in our cohort. In a prospective study conducted at two Presbyterian hospitals in New York, 79% of hospitalized patients received invasive mechanical ventilation^26^. Another study in the United Kingdom with 742 patients in 36 ICUs showed that 66.4% of patients in the severe group received invasive mechanical ventilation. Thus, despite the wide use of glucocorticoids such as dexamethasone to modulate inflammation-mediated lung injury and thereby minimize the progression of the disease to respiratory failure and death, mechanical ventilation remains the basis of clinical management of COVID-19 in severe cases, and the use of this resource is already expected for this profile of patients^27^ ^-^ ^29^.

Reported rates of acute kidney injury vary in COVID-19; however, evidence suggests that it likely affects > 20% of hospitalized patients and > 50% of patients in the ICU^30^. In our cohort, 31.8% of severe cases developed acute kidney injury during hospitalization. This frequency is similar to that reported by Cumming et al. (2020), who observed a rate of 31% in hospitalized patients^26^. Another study in the United States with 5,449 patients infected with SARS-CoV-2 admitted to the ICU observed a 36.6% incidence of acute kidney injury associated with respiratory failure, hypertension, and vasopressor medications^31^. The mechanisms of acute kidney injury in COVID-19 are not yet clear, but they likely involve multiple factors, such as direct viral infection, cytokine-mediated injury, and ischemic/hypoxic injury^32^ ^-^ ^34^.

Previous studies reported in-hospital mortality rates in severe patients admitted to the ICU, ranging from 40% to 60% at the beginning of the pandemic^35^, with a declining trend by the end of 2020^36^. In our study, the mortality rate in the severe group was 36.0%, whereas 95.3% survived in the moderate group. The period of inclusion of patients in the present study coincided with the introduction and spread of the P1 variant of concern in the country, which was responsible for an increase in the demand for ICU beds in the country, as well as an increase in the in-hospital mortality rate to values equivalent to the beginning of the pandemic^37^ ^,^ ^38^. 

Among the imaging findings, a total of 15.2% of severe patients developed pneumothorax. Although these findings are fragile, given that few patients underwent imaging exams in our study, retrospective studies of patients with COVID-19 have suggested that pneumothorax may occur in 1% of those requiring hospital admission, 2% of patients requiring ICU admission, and 1% of patients who die from infection^39^ ^-^ ^41^. More recently, the rate of barotrauma, comprising pneumothorax and pneumomediastinum, in ventilated patients was 15%^42^. Although it occurs more frequently in patients who have received invasive mechanical ventilation, studies have also shown the development of pneumothorax in patients receiving non-invasive ventilation or high-flow nasal cannulas^39^ ^,^ ^43^. Although not yet established, the mechanisms underlying the development of COVID-19-associated pneumothorax may be related to the high airway pressures provided by these respiratory support modalities and the spontaneous rupture of small, fragile airways infected with the virus^44^.

Regarding the treatment regimen, a higher frequency of corticosteroids, antibiotics, and vasopressor medication use was observed in patients with severe disease than in those with moderate. Although there is no evidence regarding the beneficial effects of ivermectin in the treatment of COVID-19^45^, this drug was used in 11.1% of cases, with no significant difference between groups. The frequency of medication use differed in most of the studies. These differences can be explained mainly by the period in which the studies were conducted since different treatment regimens were implemented throughout the pandemic as new clinical trials were published. The use of drugs such as ivermectin and hydroxychloroquine is mainly reported in studies published in 2020 ^46^ ^,^ ^47^; as our study largely involved cases hospitalized in 2021, a high frequency of use of these drugs was not observed. On the other hand, our study revealed the primary use of corticosteroid drugs (95.8%), in contrast to a French study carried out in March 2020, where this class of drugs was used in only 3.6% of hospitalized cases^48^. On the other hand, in a cohort of patients from the United States hospitalized up to December 2020, it was reported that 41.5% of hospitalized patients received at least one immunomodulatory drug^36^.

Regarding laboratory data, our findings confirm those previously published in the literature, where severe patients had greater abnormalities in hematological markers and markers related to liver and kidney function^24^. After multivariate logistic regression analysis, leukocyte count > 10,000 cells/l (OR 3.27), INR > 1.2 (OR 0.22), and urea > 48.5 mg/dL (OR 4.03) were independently associated with severe COVID-19. A study from the United States of 2,511 hospitalized patients with COVID-19 demonstrated that lymphocytopenia, eosinopenia, neutrophilia, and increased renal function markers, such as urea and creatinine, were able to predict severe COVID-19 in a prediction model using logistic regression (AUROC = 0.80)^49^. In a 2020 study, Kaeuffer et al.^50^ demonstrated a significant association between CRP, neutrophil counts, and lymphopenia and the severity of COVID-19 in a multivariate regression model that included 1,045 patients hospitalized in two French hospitals. In addition, a recent meta-analysis including 32 studies with 10,491 patients with COVID-19 demonstrated lymphopenia, thrombocytopenia, CRP, procalcitonin, D-dimer, creatine kinase, lactate dehydrogenase, AST, ALT, and creatinine levels were associated with the severity of COVID-19^51^. Leukocytes, INR, and urea are frequently used laboratory parameters in clinical practice. Using these markers at the time of hospitalization could assist in the identification of patients with worse prognosis, thus favoring the design of strategies for the clinical management of these patients.

The study design, recruitment period, circulation of different variants of the new coronavirus, and quality of the extracted data may explain the differences in the factors associated with the severity of COVID-19 in different studies. Our study has limitations, among which we can highlight the fact that the study was carried out in only two public hospitals in Northeast Brazil, which may limit the generalization of the results to other centers due to differences in the socioeconomic and demographic characteristics of the population. Second, excluding some variables from the regression model due to incomplete information may have compromised the development of a more robust prediction model. 

However, this study has several strengths. First, our study is one of the first in Brazil to investigate hospitalized patients using a large number of analyzed variables. Second, there are few published studies, including patients who became infected during the second wave of the pandemic when the P1 variant was mainly circulating in Brazil. Third, we used robust analyses to identify the potential predictors of COVID-19 severity, which may be useful in clinical practice.

This study identified the clinical and laboratory factors associated with the severity of COVID-19 using data from patients hospitalized in two reference centers in Northeast Brazil. Altered laboratory parameters of leukocytes, INR, and urea can predict the risk of severe COVID-19 in hospitalized patients. These parameters may be potential markers for the early identification of patients at a higher risk of complications, which may contribute to better management of these patients and improve survival.

## SUPPLEMENTARY MATERIAL

**SUPPLEMENTARY TABLE 1:** Reference range of laboratory indicators.

Indicator	Unit	Reference range
Leukocytes	Absolute value/µL	4000 - 10000
Lymphocytes	Absolute value/µL	1400 - 3150
Neutrophils	Absolute value/µL	2800 - 5250
Monocytes	Absolute value/µL	100 - 400
Platelets	Absolute value/mm³	150 - 450 × 10³
Blood glucose	mg/dL	< 140
CRP	mg/L	0.0 - 6.5
Ferritin	µg/L	30 - 400
D-dimer	mg/L	< 0.5
Troponin T	ng/ml	0.0 - 0.04
APTT	seconds	25 - 34
Prothrombin time	seconds	< 12.8
INR	-	0.8 - 1.2
Total bilirubin	mg/dL	0.0 - 1.20
Direct bilirubin	mg/dL	0.0 - 0.4
Indirect bilirubin	mg/dL	0.0 - 0.8
AST	U/L	5 - 50
ALT	U/L	7 - 56
GGT	U/L	8 - 61
Creatinine	mg/dL	0.5 - 1.5
Urea	mg/dL	16.6 - 48.5

**ALT:** alanine aminotransferase; **APTT:** activated partial thromboplastin time; **AST:** aspartate aminotransferase; **CRP:** C-reactive protein; **GGT:** gamma-glutamyl transferase; **INR:** international normalized ratio.
